# Präklinisches Management bei Traumapatienten und die zunehmende Zahl von Helikopter-Rettungstransporten: Eine epidemiologische Studie des TraumaRegister DGU®

**DOI:** 10.1007/s00113-023-01337-6

**Published:** 2023-07-03

**Authors:** Amelie Deluca, Christian Deininger, Florian Wichlas, Andreas Traweger, Rolf Lefering, Ernst J. Mueller

**Affiliations:** 1 Institute of Tendon and Bone Regeneration, Spinal Cord Injury & Tissue Regeneration Center Salzburg, Paracelsus Medical University, Strubergasse 21, 5020 Salzburg, Österreich; 2 Department of Trauma Surgery, KABEG-Klinikum Klagenfurt a.W., Klagenfurt, Österreich; 3 Department of Orthopedics and Traumatology, Salzburg University Hospital, Salzburg, Österreich; 4 Institute for Research in Operative Medicine (IFOM), University Witten/Herdecke, Cologne, Deutschland

**Keywords:** Traumaregister, Präklinischen Behandlung, Notfallversorgung, Transportmodalitäten, Vergleichsstudie, Trauma register, Prehospital management, Emergency medicine, Modes of transport, Comparison study

## Abstract

**Hintergrund/Ziele:**

Vergleich der präklinischen Behandlungsmodalitäten und Interventionsschemata für schwer traumatisierte Patienten mit vergleichbaren Verletzungsmustern zwischen Österreich und Deutschland.

**Patienten und Methoden:**

Diese Analyse basiert auf Daten aus dem TraumaRegister DGU®. Die Daten umfassten schwer verletzte Traumapatienten mit einem Injury Severity Score (ISS) ≥ 16, einem Alter ≥ 16 Jahre und primärer Aufnahme in ein österreichisches (*n* = 4186) oder deutsches (*n* = 41.484) Level I Trauma Center (TC) von 2008 bis 2017. Untersuchte Endpunkte umfassten präklinische Zeiten und durchgeführte Eingriffe bis zur endgültigen Krankenhauseinweisung.

**Ergebnisse:**

Die kumulierte Zeit für den Transport vom Unfallort zum Krankenhaus unterschied sich nicht signifikant zwischen den Ländern (62 min in AUT, 65 min in GER). Insgesamt wurden 53 % aller Traumapatienten in AUT mit einem Hubschrauber ins Krankenhaus transportiert, verglichen mit 37 % in GER (*p* < 0,001). Die Intubationsrate – 48 % in beiden Ländern, die Anzahl platzierter Thoraxdrainagen (5,7 % GER, 4,9 % AUT) und die Häufigkeit der verabreichten Katecholamine (13,4 % GER, 12,3 % AUT) waren vergleichbar (Φ = 0,00). Die hämodynamische Instabilität (systolischer Blutdruck (BP) ≤ 90 mmHg) bei Ankunft im TC war in AUT höher (20,6 % vs. 14,7 % bei GER; *p* < 0,001). In AUT wurden im Median 500 ml Flüssigkeit verabreicht, während in GER 1000 ml infundiert wurden (*p* < 0,001). Die demografischen Daten der Patienten zeigten keinen Zusammenhang (Φ = 0,00) zwischen beiden Ländern, und die Mehrheit der Patienten erlitt ein stumpfes Trauma (96 %). ASA-Score von 3–4 betrug 16,8 % in Deutschland (GER) gegenüber 11,9 % in Österreich (AUT).

**Fazit:**

In AUT wurden deutlich mehr Helikopter-EMS-Transporte (HEMS) durchgeführt. Die Autoren schlagen vor, eine internationale Richtlinie zu implementieren, um das HEMS-System explizit nur für Traumapatienten a) für die Rettung/Versorgung von verunfallten oder in lebensbedrohlichen Situationen befindlichen Personen, b) für den Transport von Notfallpatienten mit ISS > 16, c) für den Transport von Rettungs- oder Bergungspersonal in schwer zugängliche Regionen oder d) für den Transport von Arzneimitteln, insbesondere Blutprodukten, Organtransplantaten oder Medizinprodukten einzusetzen.

**Graphic abstract:**

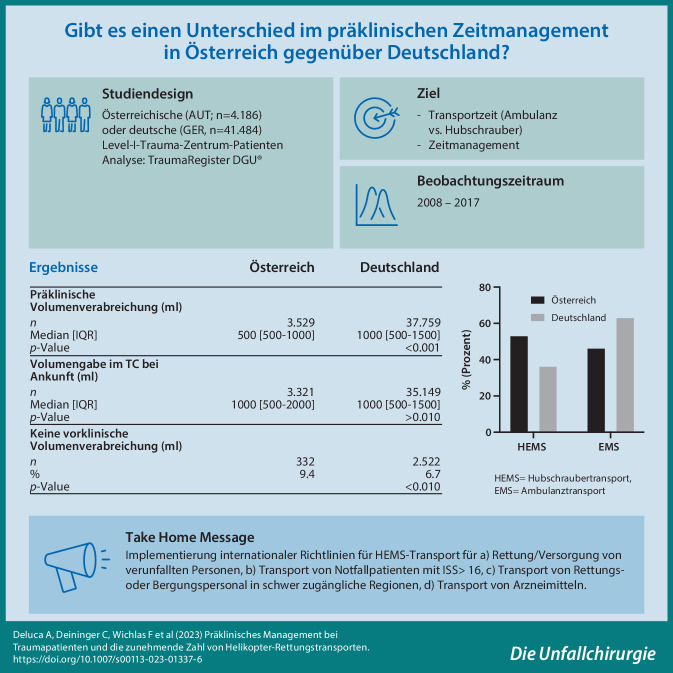

## Abstract (en)

**Background/objective:**

To compare the prehospital treatment modalities and intervention regimens for major trauma patients with comparable injury patterns between Austria and Germany.

**Patients and methods:**

This analysis is based on data retrieved from the TraumaRegister DGU®. Data included severely injured trauma patients with an injury severity score (ISS) ≥ 16, an age ≥ 16 years, and who were primarily admitted to an Austrian (*n* = 4186) or German (*n* = 41,484) level I trauma center (TC) from 2008 to 2017. Investigated endpoints included prehospital times and interventions performed until final hospital admission.

**Results:**

The cumulative time for transportation from the site of the accident to the hospital did not significantly differ between the countries (62 min in Austria, 65 min in Germany). Overall, 53% of all trauma patients in Austria were transported to the hospital with a helicopter compared to 37% in Germany (*p* < 0.001). The rate of intubation was 48% in both countries, the number of chest tubes placed (5.7% Germany, 4.9% Austria), and the frequency of administered catecholamines (13.4% Germany, 12.3% Austria) were comparable (Φ = 0.00). Hemodynamic instability (systolic blood pressure, BP ≤ 90 mmHg) upon arrival in the TC was higher in Austria (20.6% vs. 14.7% in Germany; *p* < 0.001). A median of 500 mL of fluid was administered in Austria, whereas in Germany 1000 mL was infused (*p* < 0.001). Patient demographics did not reveal a relationship (Φ = 0.00) between both countries, and the majority of patients sustained a blunt trauma (96%). The observed ASA score of 3–4 was 16.8% in Germany versus 11.9% in Austria.

**Conclusion:**

Significantly more helicopter EMS transportations (HEMS) were carried out in Austria. The authors suggest implementing international guidelines to explicitly use the HEMS system for trauma patients only a) for the rescue/care of people who have had an accident or are in life-threatening situations, b) for the transport of emergency patients with ISS > 16, c) for transportation of rescue or recovery personnel to hard to reach regions or, d) for the transport of medicinal products, especially blood products, organ transplants or medical devices.

Um die bestmögliche medizinische Versorgung fortan gewährleisten zu können, werden internationale Traumaregister ausgewertet und relevante Informationen untereinander verglichen. Dies erlaubt es, stets den Überblick über Behandlungsmethoden aber auch Verbesserungen für schwer traumatisierte Patienten im Blickwinkel zu behalten.

## Einleitung

Traumaregistervergleiche sind eine praktikable Methode zur Qualitätskontrolle, einschließlich der Identifizierung und detaillierten Analyse von Untergruppen zwischen Ländern [1]. Sie ermöglichen ferner eine Verbesserung der Versorgungsqualität [2–4], obwohl internationale Vergleiche zwischen Traumasystemen noch selten sind und es an einem einheitlichen Ansatz mangelt. Die Zuordnung der präklinischen sowie der institutionellen Leistungen zwischen den Ländern unterliegt einer sorgfältigen Patientenauswahl mit angemessenen Anpassungen in Fallmix- und funktionellen Vorhersagemodellen [5]. Diese Studie vergleicht präklinische Eingriffe in Österreich (AUT) und Deutschland (GER) anhand der Einträge im TraumaRegister DGU® (TR-DGU) mit Fokus auf polytraumatisierte Patienten. Der Vorteil dieser Studie ist die Verfügbarkeit von Dateneinträgen aus AUT und GER in die gemeinsame TR-DGU-Datenbank, die einen direkten Datenvergleich ermöglicht.

Österreich und Deutschland teilen eine große Grenze mit ähnlichen Rettungsdiensten (EMS), unterscheiden sich jedoch in ihren geografischen Umgebungen. Beide Länder setzen bei der Versorgung von Traumapatienten auf zwei unterschiedliche Transportsysteme: Helikopter- oder Krankentransport mit/ohne Arztbegleitung. Eine Vergleichsevaluation des österreichischen und des deutschen präklinischen Traumasystems wurde soweit einsehbar, noch nicht durchgeführt. Daher wurde für diese Studie ein vergleichender Ansatz gewählt, der darauf abzielt, Unterschiede im präklinischen Management zu ermitteln und Behandlungsschemata zu optimieren.

Konkret ging es in dieser Studie um vier Fragen:

1. Gibt es einen Unterschied im präklinischen Zeitmanagement in Österreich gegenüber Deutschland?
2. Unterscheidet sich die Transportart zu Level I Trauma Centers (TC) zwischen den Ländern?
3. Gibt es Unterschiede bei den präklinischen Eingriffen in den einzelnen Ländern?
4. Gibt es Unterschiede in der Patientendemografie zwischen Deutschland und Österreich?

## Methoden

### TraumaRegister DGU®

Das TraumaRegister DGU® ist eines der größten Register seiner Art weltweit und wurde 1993 gegründet. Ziel dieser multizentrischen Datenbank ist eine pseudonymisierte und standardisierte Dokumentation schwer verletzter Patienten. Die Datenerhebung erfolgt prospektiv in 4 aufeinanderfolgenden Zeitphasen vom Unfallort bis zur Entlassung aus dem Krankenhaus: A) präklinische Phase, B) Notaufnahme und Erstoperation, C) Intensivstation und D) Entlassung. Die Dokumentation umfasst detaillierte Informationen zu Demografie, Verletzungsmuster, Komorbiditäten, prä- und stationärem Management, Kurs auf der Intensivstation, relevanten Laborbefunden, einschließlich Daten zur Transfusion, und dem Outcome jedes Patienten.

Die Infrastruktur für Dokumentation, Datenmanagement und Datenanalyse wird von der AUC – Akademie der Unfallchirurgie GmbH, einem der Deutschen Gesellschaft für Unfallchirurgie angeschlossenen Unternehmen, bereitgestellt. Die wissenschaftliche Leitung liegt beim Ausschuss für Notfallmedizin, Intensivmedizin und Traumamanagement (Sektion NIS) der Deutschen Gesellschaft für Unfallchirurgie. Die teilnehmenden Krankenhäuser übermitteln ihre Daten pseudonymisiert über eine webbasierte Anwendung in eine zentrale Datenbank. Die wissenschaftliche Datenanalyse ist nach einem in der Publikationsrichtlinie der TR-DGU definierten Peer-Review-Verfahren zugelassen.

Die teilnehmenden Krankenhäuser befinden sich hauptsächlich in Deutschland (90 %), aber auch eine zunehmende Zahl von Krankenhäusern anderer Länder trägt bei (z. B. Österreich, Belgien, China, Finnland, Luxemburg, Slowenien, Schweiz, Niederlande und Vereinigte Arabische Emirate). Derzeit werden etwa 30.000 Fälle/Jahr aus mehr als 650 Krankenhäusern in die Datenbank aufgenommen.

Die Teilnahme am TR-DGU ist freiwillig. Für Krankenhäuser, die dem TR-DGU angeschlossen sind, ist jedoch aus Gründen der Qualitätssicherung die Eingabe zumindest eines Basisdatensatzes verpflichtend. Auf Basis dieser Daten können Aussagen zur Versorgungsqualität definiert und medizinische Behandlungsmethoden auf ihre Wirksamkeit untersucht werden.

Die vorliegende Studie entspricht den Publikationsrichtlinien des TraumaRegisters DGU® und ist als TR-DGU-Projekt-ID 2017-031 registriert.

### Patienten und Datenerfassung

Diese Studie ist eine retrospektive Analyse von Traumapatienten in AUT und GER, die sich auf die präklinische Zeit, den Transportmodus und die Eingriffe konzentriert, die von Ärzten im präklinischen Setting durchgeführt werden. Alle Daten wurden aus dem TraumaRegister DGU® (TR-DGU) abgerufen. Patientendaten des TR-DGU von 2008–2017 wurden analysiert. Daten vor 2008 wurden aufgrund der begrenzten Anzahl teilnehmender Krankenhäuser in Österreich ausgeschlossen. Neunundzwanzig Traumazentren (TZ) aus Österreich und nahezu alle deutschen TZ (90 %) steuerten Daten zum TR-DGU bei.

Einschlusskriterien: Erstaufnahme (d. h. keine Verlegungen/keine vorzeitige Verlegung < 48 h), Alter ≥ 16 Jahre und Injury Severity Score (ISS) ≥ 16 Punkte. Die Analyse beschränkte sich auf österreichische und deutsche Level-I-Traumazentren, die dem TraumaNetzwerk (TNW) unterstellt sind.

Der Vergleich österreichischer und deutscher Patientendaten basierte auf folgenden Kriterien:

- Altersgruppe (stratifiziert: 16–59; 60–69; 70–79; ≥ 80 Jahre),
- Geschlecht (männlich/weiblich),
- identisches Muster relevanter Verletzungen (AIS ≥ 3) in 4 anatomischen Körperregionen (Kopf, Thorax, Abdomen, Extremitäten),
- Traumamechanismus (stumpf/durchdringend),
- Schädel-Hirn-Trauma (TBI),
- Prätrauma-ASA-Score,
- Verkehrsunfall (ja/nein),
- hämodynamische Instabilität vor Ort (d. h. anfänglicher systolischer Blutdruck (BP) ≤ 90 mm Hg),
- Transportmittel: Krankenwagen mit Arzt, Helikopter mit Arzt.

Der Schwerpunkt dieser Studie lag auf präklinischen Zeiten und durchgeführten Eingriffen bis zur Übernahme der Versorgung des Patienten durch die Notaufnahme des Krankenhauses.

### Statistische Analyse

Alle Parameter aus dem TR-DGU, einschließlich der Daten von österreichischen und deutschen TC, wurden aus derselben Datenbank bezogen. Alle Vergleiche basieren auf tatsächlichen Einträgen, und es wurden keine Imputationen für Patienten mit fehlenden Daten durchgeführt.

Die TR-DGU verwendet die Version AIS 2005/Update 2008 der Abbreviated Injury Scale (AIS) in einer reduzierten Version mit 450 Codes und Online-Hilfesystemen zur Codierung. Die statistische Analyse wurde unter Verwendung von Statistical Package for the Social Sciences (SPSS; Version 24, IBM Inc., Armonk, NY, USA) und GraphPad Prism 9.0.0 (San Diego, CA, USA) durchgeführt.

Kategoriale Daten werden in Prozent dargestellt. Median und Interquartilbereich (IQR) werden für schiefe Daten und andernfalls mit Standardabweichung (SD) gemittelt. Das statistische Signifikanzniveau wurde daher auf *p* < 0,01 festgelegt (Mann-Whitney-U-Test, Chi-Quadrat-Test). Für das Maß der Assoziation zwischen dichotomen Variablen wurde der Phi-Koeffizient (Φ) verwendet, um die Stärke der Beziehung zu interpretieren (−1: perfekte negative Beziehung, 0: keine Beziehung, 1: perfekte positive Beziehung). Die Ergebnisse sollten in erster Linie im Hinblick auf ihre klinische Relevanz interpretiert werden, da die große Stichprobengröße auch bei geringfügigen Unterschieden zu einer formalen Signifikanz führen würde, weshalb für statistische Berechnungen der Phi-Koeffizient (Φ) verwendet wurde.

## Resultate

### Zeitmanagement vor dem Krankenhausaufenthalt

**Figure 1:**
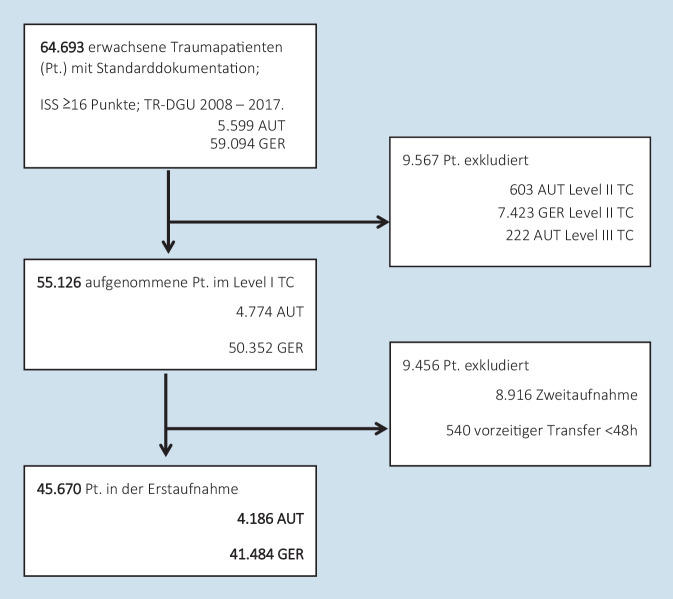

Während des beobachteten Studienzeitraums erfüllten 4186 österreichische (AUT) und 41.484 deutsche (GER) Patienten die Einschlusskriterien, die die gesamte Studienpopulation repräsentieren (Abb. 1).

Die benötigte Gesamtzeit für den Transport der Patienten vom Unfallort zum Krankenhaus unterschied sich nicht signifikant zwischen Österreich und Deutschland (*p* > 0,01) mit einem Median von 62 min und einem Interquartilbereich (IQR) von 48–80 min vs. 65 min und IQR von 50–85 min; jeweils 3 min Unterschied. Die mediane Vor-Ort-Zeit, die angibt, wie viel Zeit das Rettungsteam am Unfallort benötigte, war in Österreich mit 25 min kürzer als in Deutschland mit 28 min (*p* > 0,01). Die Transportzeit zum TC betrug 16 min in Österreich und 17 min in Deutschland (Tab. 1). Alle Transportzeiten unterschieden sich nicht signifikant zwischen den beiden Ländern (*p* > 0,01).

**Table 1:** 

	Austria	Germany
*Gesamtzeit von der Unfallstelle bis zum TC (min)*		
*n*	2954	30.684
Median [IQR]	62 [48–80]	65 [50–85]
*p*-Value	–	> 0,010
*Einsatzzeit (min)*		
*n*	2546	23.886
Median [IQR]	25 [17–35]	28 [19–40]
*p*-Value	–	> 0,010
*Transportzeit zum TC (min)*		
*n*	2613	25.986
Median [IQR] *p*-Value	16 [11–23]	17 [11–24] > 0,010

### Transportmittel

Der Transport zu TC der Stufe I erfolgte mit oder ohne Arzt. Insgesamt wurden 95,1 % der Transporte, HEMS (Helikoptertransport) und EMS (Krankentransport), in Österreich ärztlich begleitet, was nahezu gleichwertig ist mit arztbegleiteten Transporten in Deutschland, 95,9 % (*p* = 0,011).

**Figure 2:**
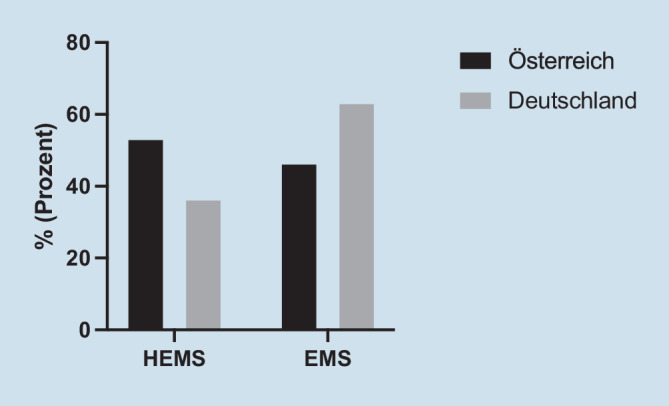

Ein deutlicher Unterschied ist bei der Transportart zu beobachten. In Österreich wurden 53,4 % der Traumapatienten per helikoptergestütztem Rettungsdienst in die Notaufnahme transportiert, verglichen mit 36,6 % in Deutschland (*p* < 0,001). In Österreich wurden die Patienten in 46,6 % der Fälle per Bodenrettung ins Krankenhaus transportiert, in Deutschland in 63,4 % (Abb. 2).

### Präklinische Eingriffe

**Figure 3:**
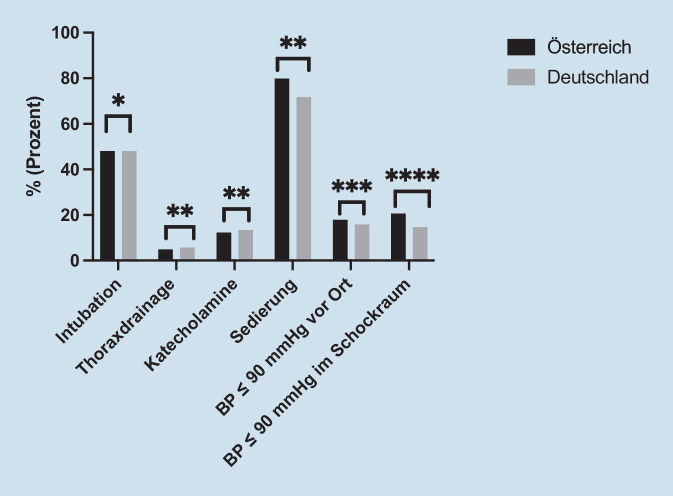

Die präklinische Intubation wurde in beiden Ländern mit 48,0 % (Φ = 0,00) in gleicher Häufigkeit durchgeführt. Der Anteil der platzierten Thoraxdrainagen lag in Deutschland bei 5,7 % im Vergleich zu 4,9 % in Österreich (Φ = 0,01). Katecholamine wurden 12,3 % der Patienten in Österreich vs. 13,4 % in Deutschland verabreicht (Φ = 0,01). Ein größerer Unterschied zwischen den Ländern ist in der Menge der erhaltenen Sedierung/Analgetika zu beobachten. Eine Sedierung wurde in Österreich bei 80,0 % und in Deutschland bei 71,8 % der Patienten durchgeführt (Φ = 0,01). Eine weitere Unterscheidung gilt für die empfundene hämodynamische Stabilität am Unfallort: Ein systolischer Blutdruck (BD) ≤ 90 mm Hg wurde bei 17,9 % der österreichischen Patienten und bei 15,9 % der Deutschen beobachtet (Φ = 0,02). Zum Zeitpunkt des Eintreffens im TC waren 20,6 % der österreichischen Patienten hämodynamisch instabil, mit einem systolischen Blutdruck ≤ 90 mm Hg gegenüber 14,7 % in Deutschland (Φ = 0,05) (Abb. 3). Insgesamt lässt sich zwischen den Ländern kein Zusammenhang zwischen den oben genannten präklinischen Eingriffen feststellen.

Ein signifikanter Unterschied gilt für das gezielte präklinische Flüssigkeitsmanagement: Traumapatienten wurden in Österreich im Median 500 ml (IQR: 500–1000 ml) Flüssigkeit verabreicht, in Deutschland im Median 1000 ml (IQR: 500–1500 ml) (*p* < 0,001). Bei der Ankunft in der Notaufnahme (ED) erhielten die Patienten in Österreich (IQR: 500–2000) und Deutschland (IQR: 500–1500) 1000 ml Flüssigkeit (Tab. 2).

**Table 2:** 

	Austria	Germany
*Präklinische Volumenverabreichung (ml)*		
*n*	3529	37.759
Median [IQR]	500 [500–1000]	1000 [500–1500]
*p*-Value	–	< 0,001
*Volumengabe im TC bei Ankunft (ml)*		
*n*	3321	35.149
Median [IQR]	1000 [500–2000]	1000 [500–1500]
*p*-Value	–	> 0,010
*Keine vorklinische Volumenverabreichung (ml)*		
*n*	332	2522
%	9,4	6,7
*p*-Value	–	< 0,010

### Patientendemografie

Ein stumpfes Trauma erlitten 96,0 % der gesamten Studienpopulation ohne Unterschied zwischen den beiden Teilpopulationen. Nach dem Verletzungsmechanismus meldete Deutschland eine höhere Rate an Verkehrsunfällen mit Beteiligung von Autos, Motorrädern und Fahrrädern. Das Alter der Patienten unterschied sich nicht zwischen Deutschland, 51,3 ± 20,9 Jahre mit einem mittleren ISS von 28,0 ± 11,9 Punkten, und Österreich, mittleres Alter: 50,1 ± 20,3 Jahre, mittlerer ISS: 27,9 ± 11,2 Punkte. Der beobachtete Score 3–4 der American Society of Anesthesiologists (ASA) (http://www.asahq.org), der sich auf Patienten mit einer schweren Erkrankung bezieht, lag in Deutschland bei 16,8 % gegenüber 11,9 % in Österreich. Dies entspricht einem Unterschied von 4,9 %, wobei sich die Prävalenz relevanter Verletzungen innerhalb der 4 Hauptkörperregionen (AIS ≥ 3) nicht unterschied. Für alle anderen analysierten Parameter waren die Häufigkeiten zwischen Österreich und Deutschland vergleichbar, und insgesamt zeigte der Phi(Φ)-Koeffizient keinen Zusammenhang zwischen den verschiedenen demografischen Daten (Tab. 3).

**Table 3:** 

	Österreich *n* = 4186	Deutschland *n* = 41.484	Φ (Phi)
*Stumpfes Trauma (%)*	95,3	95,9	0,09
*Weiblich (%)*	25,3	28,3	0,02
*Age: 16–59 years (%)*	66,1	63,7	0,01
*Age: 60–69 years (%)*	12,6	12,2	0,01
*Age: 70–79 years (%)*	12,2	13,7	0,01
*Age: ≥* *80 years (%)*	9,1	10,5	0,01
*Mean Age* *±* *SD*	50,1 ± 20,3	51,3 ± 20,9	–
*Mean ISS* *±* *SD*	27,9 ± 11,2	28,0 ± 11,9	–
*ASA 3–4 (%)*	11,9	16,8	0,04
*AIS, Kopf ≥* *3 (%)*	56,5	56,9	0.00
*AIS, Thorax ≥* *3 (%)*	54,8	56,7	0,01
*AIS, Abdomen ≥* *3 (%)*	17,7	16,1	0,01
*AIS, Pelvis/Extremitäten ≥* *3 (%)*	30,8	32,4	0,01
*TBI, isolated (%)*	16,7	14,7	0,02
*Unfallmechanismus (%)*			
Auto/LKW	20,0	22,9	0,02
Motorrad	12,2	13,1	0,01
Fahrrad	7,4	8,9	0,02
Fußgänger	7,3	7,6	0,00
Niedriger Fall < 3 m Höhe	17,2	18,0	0,01
Hoher Fall > 3 m Höhe	20,6	19,0	0,01
Stumpfer Schlag	4,7	2,4	0,04
Schussverletzung	1,5	0,8	0,02
Stichverletzung	1,1	1,3	0,01
Sonstiges	5,3	4,7	0,01

## Diskussion

Unsere Analyse zeigt, dass das präklinische Management schwer traumatisierter Patienten zwischen Österreich und Deutschland nicht viele Unterschiede aufweist. Die beobachteten Ergebnisse, basierend auf Registerdaten, umfassen das präklinische Management von Patienten.

Die mediane Gesamttransportzeit und die Einsatzzeit des Rettungsdienstes waren in Österreich um 3 min kürzer. Der Effekt dieses Unterschieds ist auf das Transportmittel zurückzuführen (53,4 % Helikoptertransport in Österreich gegenüber 36,6 % in Deutschland). Mehr als die Hälfte (53,4 %) aller österreichischen Traumapatienten wurden mit einem Helikopter in ein TC transportiert, verglichen mit 36,6 % in Deutschland (*p* < 0,001). EMS in Österreich werden stark durch ihre geografische Ausrichtung und Bevölkerungsverschiebungen während der touristischen Hauptsaison (Sommer und Winter) beeinflusst. 50 % der österreichischen Bevölkerung leben in Kleinstädten oder Dörfern, die über das ganze Land verstreut sind, wodurch ein Stadt-Land-Split (50:50) entsteht. Daraus ergibt sich die Notwendigkeit von Anpassungen der EMS-Strategien auf Basis einzelner Länderseiten. In ländlichen Gebieten sind die Transportzeiten viel länger, da die Entfernungen zu medizinischen Einrichtungen mehr als 60 km betragen können. Daher werden ärztlich besetzte Helikopter überwiegend in ländlichen Gebieten eingesetzt, insbesondere in alpinen Regionen, wo die ausschließliche Nutzung von Bodentransportmitteln schwierig oder sogar unmöglich ist [8]. Derzeit gibt es 38 Hubschrauber-EMS (HEMS) verteilt über ganz Österreich und eine Fläche von 84.000 Quadratkilometern (1:2211 Quadratkilometer) und 89 in Deutschland mit einer Fläche von 356.000 Quadratkilometern (1:4000 Quadratkilometer) [9]. Darüber hinaus ist der gesamte Westen sowie die meisten südlichen und zentralen Teile Österreichs von den Alpen bedeckt, wo einige Berge Höhen bis zu 3800 Metern erreichen, und sich daher auf ein medizinisch basiertes HEMS verlassen [8]. Ein weiterer Grund, der den hohen Helikopteranteil in Österreich erklären könnte, ist die durch den Tourismus bedingte Zunahme der Zahl der Traumapatienten. Laut http://www.austria.info wurden 2021 79,6 Mio. Übernachtungen von Touristen aus aller Welt gezählt.

Dennoch werden die potenziell vorteilhaften Auswirkungen von HEMS auf das Behandlungsergebnis und die Kosteneffizienz noch immer kontrovers diskutiert [23]. Zusammenfassend profitieren Traumapatienten von der HEMS-Rettung mit dem Überleben im Krankenhaus als Hauptergebnisparameter. Die Analyse verschiedener Untergruppen, ältere Patienten, Niedrigenergietrauma und die Schwere geringfügiger Verletzungen hatten den ausgeprägtesten Überlebensvorteil, wenn sie durch HEMS gerettet wurden [24]. Allerdings wurden nach Angaben des österreichischen (OEAMTC) und deutschen (ADAC) Automobilclubs im Jahr 2019 insgesamt 17.281 bzw. 53.967 HEMS-Transporte in die ED durchgeführt. Dies entspricht 1942 HEMS-Transporten pro 1 Mio. Einwohner in Österreich und 650 Transporten pro 1 Mio. in Deutschland. Der große Unterschied bei Hubschraubertransporten lässt sich ferner durch das allgemeine Fehlen eines regional/national einheitlichen Algorithmus von Hinweisen/Richtlinien für den Transport erklären. Wir empfehlen, das HEMS-System explizit nur für Traumapatienten einzusetzen a) zur Rettung/Versorgung von verunfallten oder in lebensbedrohlichen Situationen befindlichen Personen, b) zum Transport von Notfallpatienten mit ISS > 16, c) für Transport von Rettungs- oder Bergungspersonal in schwer zugängliche Regionen oder d) für den Transport von Arzneimitteln, insbesondere Blutprodukten, Organtransplantaten oder Medizinprodukten. Zusammenfassend erscheint die Nutzung des HEMS-Systems in Österreich im Vergleich zu Deutschland zu hoch und sollte hinsichtlich seiner Praxisrelevanz neu bewertet werden.

Darüber hinaus zeigt diese Studie eine Diskrepanz in der präklinischen Flüssigkeitsverabreichung. Ein signifikanter Unterschied zwischen den Ländern wurde für die vor Ort verabreichte Flüssigkeitsmenge beobachtet, ein Median von 500 ml in Österreich gegenüber 1000 ml in Deutschland. Dies kann auf Unterschiede in den Gewohnheiten der Flüssigkeitszufuhr zurückzuführen sein, wie sie in der Schweiz [25] und auch in der niederländischen Bevölkerung [6] zu beobachten sind, wo im Vergleich zu Deutschland 30 % weniger Flüssigkeit im präklinischen Bereich verabreicht werden. Kudoet al. erläutern, wie wichtig es ist, ein Gleichgewicht zwischen Organperfusion und Hämostase zu erreichen, das für eine optimale Flüssigkeitsreanimation bei Traumapatienten entscheidend ist. „Permissive Hypotonie“ bezieht sich auf die Behandlung von Traumapatienten durch Begrenzung der Menge an Reanimationsflüssigkeit und Aufrechterhaltung des Blutdrucks auf einem niedrigeren als dem normalen Bereich, wenn es während der akuten Verletzungsphase zu Blutungen kommt. Allerdings hat keine Studie untersucht, welche Probanden am meisten von diesem Ansatz profitieren würden, wenn Faktoren wie Alter, Verletzungsmechanismus, Umgebung oder das Vorhandensein oder Fehlen einer Hypotonie berücksichtigt werden [26]. Der Einfluss dieser Ansätze auf die Gerinnung wurde nicht ausreichend untersucht, selbst in Tierversuchen, und die Gesamtwirksamkeit der permissiven Hypotonie/hypotensiven Reanimation ist noch nicht schlüssig und erfordert weitere Forschung [10–12, 21].

Es gibt keine Evidenz zur erforderlichen Volumenschwelle für Traumapatienten [13]. Driessen et al. geben an, dass der mittlere Basenüberschuss (BE) zum Zeitpunkt der Aufnahme in die Notaufnahme trotz niedrigem Blutdruck auf eine bessere Gewebedurchblutung hindeuten könnte [14]. In Österreich kamen 20,6 % im Vergleich zu 14,7 % der Deutschen in einem hämodynamisch instabilen Zustand ins TC, verbunden mit einem systolischen Blutdruck ≤ 90 mm Hg. Die Gabe von Katecholaminen rekrutiert unbelastete Blutvolumina und beeinflusst die Aufrechterhaltung des Blutdrucks [15]. Dies könnte eine mögliche Erklärung dafür sein, warum die beobachtete hämodynamische Instabilität österreichischer Patienten, die ins TC kamen, höher war, sowie die reduzierte Menge an Flüssigkeit, die vor Ort verabreicht wurde. Weiterhin kann ausgeschlossen werden, dass die Gabe von Tranexamsäure (TXA) für den Unterschied im systolischen Blutdruck bei Ankunft im TC verantwortlich ist, da die verabreichte Menge an Tranexamsäure in beiden Ländern gleichwertig war. Insgesamt kann von den Autoren kein Algorithmus zur genauen und korrekten Menge des Flüssigkeitsvolumens im präklinischen Setting empfohlen werden, da es eine abhängige Variable von Alter, Antikoagulation, Blutverlust und beobachtetem Verletzungsmuster ist [19].

Sowohl der Verletzungsmechanismus als auch verschiedene demografische Merkmale zeigen keine Beziehung zwischen einzelnen Variablen zwischen beiden Ländern und waren ähnlich wie in anderen Studien, die mit dem TR-DGU durchgeführt wurden [4, 6, 7, 20]. Im Allgemeinen sollten die Ergebnisse in erster Linie im Hinblick auf ihre klinische Relevanz interpretiert werden, da die große Stichprobengröße, die in diese Studie eingeschlossen wurde, wahrscheinlich auch bei geringfügigen Unterschieden eine formale Signifikanz ergibt. Allerdings können Variationen beobachtet werden, wenn geriatrische Altersgruppen (≥ 70 Jahre) innerhalb eines Landes kombiniert werden. Hervorzuheben ist, dass in Deutschland mehr schwer verletzte geriatrische Patienten in TC transportiert wurden als in Österreich. Dies korreliert weiter mit einem höheren beobachteten ASA-Score [16, 17] in Deutschland mit zunehmendem Alter der Patienten. Spering et al. deuten darauf hin, dass die Anzahl der Patienten mit vorbestehenden Erkrankungen, klassifiziert nach ASA ≥ 3, mit zunehmendem Alter zunimmt. Mit zunehmendem Alter der verletzten Patienten steigt somit auch der kumulierte Anteil von ASA 3 und 4 [22]. Insgesamt wurde eine größere Anzahl geriatrischer Patienten in Deutschland in diese retrospektive Datenanalyse eingeschlossen, was mit einem höheren beobachteten ASA-Score (Klassen 3 und 4) korreliert. Dieser Klassifikation-Score umfasst Patienten mit mittelschwerer bis schwerer systemischer Erkrankung und Funktionseinschränkungen bis hin zu einer schweren systemischen Erkrankung, die eine ständige Lebensbedrohung darstellt [18].

## Limitationen

Diese retrospektive Studie wurde mit Daten aus einem großen Register durchgeführt, beinhaltet aber nicht Daten aller österreichischen TC. Daher führte die Vollständigkeit der eingereichten Daten zu Einschränkungen, da nicht alle TC in Österreich an der Studie teilnahmen. Außerdem wurde die Überprüfung der Richtigkeit der eingegebenen Daten gemäß TR-DGU nur in einer kleinen Stichprobe von Fällen durchgeführt. Die berichteten Unterschiede in den Ergebnissen könnten auf statistische Zufallsbefunde (Fehler 1. und 2. Art), Unterschiede in der Pflege und verschiedene Behandlungsprotokolle zurückzuführen sein. Trotz der Einschränkungen von Traumaregistern dient es als Mittel zur Qualitätskontrolle und hilft bei der Optimierung von Behandlungsprotokollen.

## „Conclusion“

Es gab keinen signifikanten Unterschied zwischen Österreich und Deutschland, basierend auf grundlegenden demografischen Merkmalen. Die Einsatzzeit des Rettungsdienstes war in Österreich um 3 min kürzer, was wahrscheinlich auf die vorherrschende Verwendung von HEMS zurückzuführen ist. Obwohl geografische Veränderungen und Tourismus die deutlich höhere Zahl von Hubschraubertransporten in Österreich erklären könnten, schlagen die Autoren vor, eine internationale Richtlinie zu implementieren, um das HEMS-System explizit für bestimmte Indikationen zu verwenden. Insgesamt sollte die Menge der verabreichten Flüssigkeit in der vorklinischen Umgebung dem Konzept der permissiven Hypotonie/hypotonen Reanimation folgen, insbesondere bei jüngeren Patienten, ist jedoch noch nicht schlüssig und erfordert weitere Forschung. Darüber hinaus sollten Qualitätskontrolle und Registervergleiche ein grundlegender Bestandteil der Behandlung von Traumapatienten in Krankenhausorganisationen sein.

## Fazit für die Praxis

Implementation einer internationalen Richtlinie für das HEMS-System für Traumapatienten sollte folgende Punkte berücksichtigen: Die Rettung und/oder Versorgung von Verunfallten, den Transport von hauptsächlich Notfallpatienten (ISS > 16), den Transport von Rettungs- und/oder Bergungspersonal in schwer zugänglichen Regionen und den Notfalltransport von Arzneimitteln (Blutprodukte, Organtransplantate).
